# Next generation sequence analysis for mitochondrial disorders

**DOI:** 10.1186/gm100

**Published:** 2009-10-23

**Authors:** Valeria Vasta, Sarah B Ng, Emily H Turner, Jay Shendure, Si Houn Hahn

**Affiliations:** 1 Seattle Children's Research Institute, 1900 9th Ave, Seattle, WA 98101, USA; 2 Department of Genome Sciences, University of Washington, 1705 NE Pacific St, Seattle, WA 98195, USA; 3 Department of Pediatrics, University of Washington, 4800 Sand Point Way NE, Seattle, WA 98105, USA

## Abstract

**Background:**

Mitochondrial disorders can originate from mutations in one of many nuclear genes controlling the organelle function or in the mitochondrial genome (mitochondrial DNA (mtDNA)). The large numbers of potential culprit genes, together with the little guidance offered by most clinical phenotypes as to which gene may be causative, are a great challenge for the molecular diagnosis of these disorders.

**Methods:**

We developed a novel targeted resequencing assay for mitochondrial disorders relying on microarray-based hybrid capture coupled to next-generation sequencing. Specifically, we subjected the entire mtDNA genome and the exons and intron-exon boundary regions of 362 known or candidate causative nuclear genes to targeted capture and resequencing. We here provide proof-of-concept data by testing one HapMap DNA sample and two positive control samples.

**Results:**

Over 94% of the targeted regions were captured and sequenced with appropriate coverage and quality, allowing reliable variant calling. Pathogenic mutations blindly tested in patients' samples were 100% concordant with previous Sanger sequencing results: a known mutation in Pyruvate dehydrogenase alpha 1 subunit (*PDHA1*), a novel splicing and a known coding mutation in Hydroxyacyl-CoA dehydrogenase alpha subunit (*HADHA*) were correctly identified. Of the additional variants recognized, 90 to 94% were present in dbSNP while 6 to 10% represented new alterations. The novel nonsynonymous variants were all in heterozygote state and mostly predicted to be benign. The depth of sequencing coverage of mtDNA was extremely high, suggesting that it may be feasible to detect pathogenic mtDNA mutations confounded by low level heteroplasmy. Only one sequencing lane of an eight lane flow cell was utilized for each sample, indicating that a cost-effective clinical test can be achieved.

**Conclusions:**

Our study indicates that the use of next generation sequencing technology holds great promise as a tool for screening mitochondrial disorders. The availability of a comprehensive molecular diagnostic tool will increase the capacity for early and rapid identification of mitochondrial disorders. In addition, the proposed approach has the potential to identify new mutations in candidate genes, expanding and redefining the spectrum of causative genes responsible for mitochondrial disorders.

## Background

Mitochondrial disorders are the most common group of metabolic disorders, with an estimated prevalence of 1 in 5,000 [1]. Mitochondrial disorders may present with any symptom, in any organ, at any age and with any mode of inheritance [2]. These often devastating disorders are clinically characterized by multi-system involvement with primarily progressive neurologic disease and myopathy, including both skeletal and cardiac muscle. The variability in clinical presentation and underlying causative mutations make the diagnosis very challenging, involving extensive clinical and specialized laboratory evaluation [3]. However, no reliable diagnostic screening or biomarker is available that is both sensitive and specific in all cases of mitochondrial disorders [4]. In current clinical practice, the diagnosis of mitochondrial disease relies heavily on identifying deficient activity of one or more of the mitochondrial respiratory chain enzymes, but in many cases the enzyme activity is found to be only moderately decreased or even normal, which makes the interpretation very difficult. Additionally, there are intrinsic problems in the biochemical characterization of mitochondrial disorders, such as variability in tissue manifestation, difficulty in establishing realistic normal reference ranges, inability of enzyme assays to detect some functional defects, variations in assay protocols, no uniform or standardized guidelines, and lack of widely accepted diagnostic criteria and a quality assurance scheme [5]. For these reasons, some patients may remain undiagnosed and even die of untreated disease. Early and definitive diagnosis is crucial for permitting appropriate management and accurate counseling [3]. Thus, a new simplified and reliable approach for the diagnosis of mitochondrial disorders with better accuracy and precision has been strongly advocated.

Mitochondria are cellular organelles with numerous essential functions, such as production of energy, metabolism of amino acids, fatty acids, and cofactors, and cell signaling. Their biogenesis and function is under the genetic control of mitochondrial DNA (mtDNA) and nuclear DNA. The number of mitochondrial proteins encoded by nuclear genes is estimated to be around 1,500 [6], constituting 99% of mitochondrial proteins [7]. mtDNA contains 37 genes encoding 13 respiratory chain subunits, 2 rRNAs and 22 tRNAs [8]. Because of this dual genetic control, mitochondrial disorders can originate from mutations in either mtDNA or nuclear genes that encode the organelle proteins [8].

Mutations have been found in approximately 170 nuclear genes in patients with mitochondrial disorders [6,8,9]. However, many nuclear genes causing disease are still unknown [8]. It is expected that most mitochondrial disorders are caused by mutations in nuclear genes. The nuclear genes encode the subunits of the complexes involved in oxidative phosphorylation and relative assembly factors, proteins controlling the synthesis and stability of mtDNA, mitochondria transcription and translation, biogenesis, metabolism, and signaling. While substantial progress has been made in recent years in identifying nuclear genes that are mutated in mitochondrial disorders, the key clinical challenge lies in determining which one of hundreds of genes is responsible for the disease in any given patient. Comprehensive sequencing of all nuclear genes known to be involved in mitochondrial disease would be cost-prohibitive and time consuming using traditional DNA sequencing technology. Not surprisingly, clinical tests are available for only a limited number of nuclear genes for a few conditions in which the causative genes can be predicted by the clinical phenotypes [8].

Pathogenic mutations in mtDNA are found in at least one in 5,000 affected individuals [1] and appear to be very common in the general population (>1 in 200 live births) even though these mutations show different penetrance during the lifetime of the carriers. Many of these mutations are primarily responsible for adult-onset mitochondrial disorders [1]. Mutations in nuclear genes are likely the major cause of mitochondrial disease particularly in pediatric cases [3]. Nevertheless, the pediatric prevalence of mtDNA mutations may have been underestimated because mtDNA testing is typically performed by targeted mutation analysis. This strategy cannot identify mutations beyond those targeted [10]. While mtDNA (16,569 bp) could easily be sequenced by traditional Sanger methods, this technology is inadequate to detect some mtDNA mutations that occur in a small fraction of the total mtDNA molecules. Indeed, mutated molecules of mtDNA coexist with normal mtDNA (heteroplasmy) and can be below the limit of detection of Sanger sequencing, especially in DNA extracted from blood [11].

Within the last few years, next-generation sequencing has been tested for whole genome or targeted resequencing with promising results. The new platforms allow sequencing hundreds of genes in parallel and detection of mutations or alterations with a dramatically reduced cost. Over the coming years, next-generation sequencing is highly anticipated to transition from basic research applications into clinical diagnostics [12-14]. One such opportunity is the rapid identification of mutations in diseases that can be caused by one of several genes, as with mitochondrial disorders.

Despite the fact that whole genome or exome sequencing is now possible, it is still desirable to limit the analysis to genes responsible for a certain condition for both cost benefit and time saved for analysis and interpretation. Given that most pathogenic mutations are typically located in coding regions or at intron-exon boundaries, and that it is not practical to use PCR to enrich a large number of exons, several methodologies have been developed to enrich exons of target genes as a preliminary step to next-generation sequencing [15-20]. Compared with whole genome/exome sequencing, this enables a major reduction in cost and allows higher sequence coverage over the areas of interest.

Here, we propose to develop a comprehensive clinical diagnostic tool based on sequencing the entire mtDNA genome, and the exons of previously implicated and candidate nuclear genes (Table 1) using sequence capture technology coupled to next-generation sequencing.

**Table 1:** Genes targeted for capture and sequencing

OXPHOS subunits	OXPHOS assembly factors	Enzymes	Transcription/translation	Carriers	mtDNA maintenance, mitochondria biogenesis/dynamics
**Known genes for mitochondrial disorders**					
*COX4I2*, *COX6B1*, *NDUFA1*, *NDUFA2*, *NDUFA11*, *NDUFS1*, *NDUFS2*, *NDUFS3*, *NDUFS4*, *NDUFS6*, *NDUFS7*, *NDUFS8*, *NDUFV1*, *NDUFV2*, *SDHA*, *SDHB*, *SDHC*, *SDHD*, *UQCRB*, *UQCRQ*	*ATPAF2*, *BCS1L*, *C20orf7*, *COX10*, *COX15*, *NDUFAF1NDUFAF3*, *NDUFAF4*, *SCO1*, *SCO2*, *SURF1*	*ABCB7*, *ACAT1*, *APTX*, *ASS1*, *BCKDHA*, *BCKDHB*, *CABC1*, *COQ9*, *CYCS*, *DBT*, *DLAT*, *DLD*, *ETHE1*, *FH*, *FXN*, *GFER*, *HADH*, *HADHA*, *HADHB*, *HMGCL*, *HMGCS2*, *MCCC2*, *OXCT1*, *PC*, *PCK2*, *PDHA1*, *PDHB*, *PDHX*, *PDSS1*, *PDSS2*, *PPM1B*, *PPM2C*, *PREPL*, *TAZ*	*ATXN7*, *DARS2*, *GFM1*, *LRPPRC*, *MRPS16*, *MRPS22*, *PUS1*, *RARS2*, *TSFM*, *TUFM*	*DNAJC19*, *SLC25A13*, *SLC25A15*, *SLC25A19*, *SLC25A20*, *SLC25A22*, *SLC25A3*, *SLC25A4*, *SLC3A1*, *TIMM8A*	*c10orf2*, *DGUOK*, *DNM1L*, *ETFA*, *ETFB*, *ETFDH*, *HSPD1*, *MFN2*, *MPV17*, *OPA1*, *PINK1*, *POLG2*, *RRM2B*, *SPG7*, *SUCLA2*, *SUCLG1*, *TK2*, *TYMP*, *WFS1*
**Candidate genes for mitochondrial disorders**					
*ACOT7*, *AMACR*, *ATP5A1*, *ATP5B*, *ATP5C1*, *ATP5D*, *ATP5E*, *ATP5F1*, *ATP5G1*, *ATP5G2*, *ATP5G3*, *ATP5H*, *ATP5I*, *ATP5J*, *ATP5J2*, *ATP5O*, *ATP5S*, *ATPIF1*, *BCL2L13*, *C10orf65*, *C14orf2*, *COX4I1*, *COX5A*, *COX5B*, *COX6A1*, *COX6A2*, *COX6C*, *COX7A1*, *COX7A2*, *COX7B*, *COX7B2*, *COX7C*, *COX8A*, *COX8C*, *CYC1*, *DCI*, *FOXRED1*, *GAD1*, *GBAS*, *GNPAT*, *GPAM*, *HPDL*, *IVD*, *MGST3*, *MRRF*, *NDUFA10*, *NDUFA12*, *NDUFA13*, *NDUFA3*, *NDUFA4*, *NDUFA5*, *NDUFA6*, *NDUFA8*, *NDUFA9*, *NDUFAB1*, *NDUFB1*, *NDUFB10*, *NDUFB11*, *NDUFB2*, *NDUFB3*, *NDUFB4*, *NDUFB5*, *NDUFB6*, *NDUFB7*, *NDUFB8*, *NDUFB9*, *NDUFC1*, *NDUFC2*, *NDUFS5*, *NDUFV3*, *NIPSNAP1*, *NIPSNAP3A*, *NIPSNAP3B*, *NPL*, *PAH*, *PHYH*, *PNPT1*, *PRO1853*, *RNMTL1*, *TMEM22*, *UQCR*, *UQCRC1*, *UQCRC2*, *UQCRFS1*, *UQCRH*, *USMG5*	*ATPAF1*, *COX11*, *COX17*, *COX18*, *COX19*, *ECSIT*	*COQ10B*, *COQ3*, *COQ4*, *COQ5*, *COQ6*, *COQ7*, *PDK1*, *PDK2*, *PDK3*, *PDK4*, *PDP2*, *PDPR*	*AARS2*, *CARS2*, *FARS2*, *IARS2*, *KARS*, *LARS2*, *MARS2*, *MRPL1*, *MRPL10*, *MRPL11*, *MRPL12*, *MRPL13*, *MRPL14*, *MRPL15*, *MRPL16*, *MRPL17*, *MRPL18*, *MRPL19*, *MRPL2*, *MRPL20*, *MRPL21*, *MRPL22*, *MRPL23*, *MRPL24*, *MRPL27*, *MRPL28*, *MRPL3*, *MRPL30*, *MRPL32*, *MRPL33*, *MRPL34*, *MRPL35*, *MRPL36*, *MRPL37*, *MRPL38*, *MRPL39*, *MRPL4*, *MRPL40*, *MRPL41*, *MRPL42*, *MRPL43*, *MRPL44*, *MRPL45*, *MRPL46*, *MRPL47*, *MRPL49*, *MRPL50*, *MRPL51*, *MRPL52*, *MRPL53*, *MRPL54*, *MRPL55*, *MRPL9*, *MRPS10*, *MRPS11*, *MRPS12*, *MRPS14*, *MRPS15*, *MRPS17*, *MRPS18A*, *MRPS18B*, *MRPS18C*, *MRPS2*, *MRPS21*, *MRPS23*, *MRPS24*, *MRPS25*, *MRPS26*, *MRPS27*, *MRPS28*, *MRPS29*, *MRPS30*, *MRPS31*, *MRPS33*, *MRPS34*, *MRPS35*, *MRPS36*, *MRPS5*, *MRPS6*, *MRPS7*, *MRPS9*, *MTG1*, *NARS2*, *NRF1*, *PARS2*, *RSPH9*, *SARS2*, *TARS2*, *TFAM*, *TFB1M*, *TFB2M*, *WARS2*, *YARS2*	*GRPEL1*, *HSPA9*, *PAM16*, *SAMM50*, *SLC25A1*, *SLC25A10*, *SLC25A11*, *SLC25A12*, *SLC25A14*, *SLC25A16*, *SLC25A17*, *SLC25A18*, *SLC25A2*, *SLC25A21*, *SLC25A23*, *SLC25A24*, *SLC25A25*, *SLC25A26*, *SLC25A29*, *SLC25A30*, *SLC25A31*, *SLC25A32*, *SLC25A33*, *SLC25A34*, *SLC25A35*, *SLC25A36*, *SLC25A38*, *SLC25A39*, *SLC25A40*, *SLC25A42*, *SLC25A43*, *SLC25A44*, *SLC25A45*, *SLC25A46*, *SLC25A5*, *SLC25A6*, *TIMM10*, *TIMM13*, *TIMM17A*, *TIMM17B*, *TIMM22*, *TIMM23*, *TIMM44*, *TIMM50*, *TIMM8B*, *TIMM9*, *TOMM20*, *TOMM22*, *TOMM34*, *TOMM40*, *TOMM40L*, *TOMM7*, *TOMM70A*, *UCP1*, *UCP2*, *UCP3*, *VDAC1*, *VDAC2*, *VDAC3*	*HSPE1*, *MFN1*,

## Methods

Positive control patient samples were obtained as anonymous samples from Seattle Children's Hospital; these were leftover specimens after routine standard clinical testing. Mutations in these samples were previously identified by clinical tests using traditional sequencing. Patient 1 was Caucasian and Patient 2 was of Caucasian-Native American origin. Human DNA from one HapMap individual was obtained from Coriell Repositories (NA18517, Yoruba ancestry). We used 10 μg of DNA per individual for these studies.

Custom programmable arrays (Agilent Technologies Inc.) were designed with 60-mer oligonucleotide probes complementary to the sequences to be captured. The target consisted of the entire mtDNA genome and coding sequences within >3,500 exons of 362 nuclear genes for proteins involved in mitochondrial function, for an aggregate target size of approximately 0.6 Mb (Table 1) excluding repetitive regions. The Consensus CDS (CCDS) database was utilized to obtain the exon coordinates for probe design. Due to discrepancies between identifiers used by our group and CCDS or because CCDS is not comprehensive, a small number of genes were inadvertently excluded from the array design process. These included, for example, Polymerase gamma 1 (*POLG1*), a nuclear gene involved in mitochondrial disorders [21] (annotated as POLG in CCDS), and highlights the importance of careful review in the design process.

As there are approximately 244,000 programmable oligos on the custom arrays used here, the targeted sequences were 'tiled' at a very high density (that is, 40 probes per 100-bp interval; probe sequences are available upon request). To construct an *in vitro *shotgun sequencing library, genomic DNA was sheared by nebulization and universal adaptor oligonucleotides were ligated and then amplified using the Illumina protocol [22]. After this step, in order to enrich for the specific target exons and mtDNA, the amplified shotgun libraries were hybridized to the capture array as described in [23]. After washing to remove unhybridized material, captured molecules were recovered by heat-based elution and subjected to PCR amplification. The target-enriched shotgun libraries were quantified (NanoDrop Products, Wilmington, DE, USA), and then subjected to deep sequencing on an Illumina Genome Analyzer, GAII. One lane of the flow cell was used for each sample. Read-lengths of up to 36 bp were obtained with per-base accuracies on the order of 99%. The sequence reads were aligned to the human reference genome, first using the standard Illumina package (ELAND). After removal of all but one of the reads mapping with identical coordinates and orientation (potential PCR duplicates), the remaining reads were remapped using the MAQ software package [24]. Consensus calls for variant identification were also carried with MAQ.

In order to assess the significance of new variants found in the study, we analyzed the non-synonymous single nucleotide substitutions with PolyPhen (Polymorphism Phenotyping), a tool that predicts the possible impact of an amino acid substitution on the structure and function of a human protein using physical and comparative considerations [25].

## Results

### Depth of coverage across targeted regions

A single lane of an Illumina flow cell was used for each sample, producing 356 Mb, 297 Mb, and 333 Mb for the HapMap, patient 1 and patient 2 samples, respectively, that mapped to the human genome with the Illumina ELAND software (36 bp, single-end reads; Table 2). Of these, 17%, 35% and 30% mapped to the approximately 0.6 Mb of targeted regions in the nuclear genome, and 37%, 20% and 27% mapped to the 16.6-kb mitochondrial genome. Although mtDNA was represented on the capture array at an equivalent density to nuclear genes, its high copy number is likely responsible for its significantly greater degree of enrichment. After removal of potential PCR duplicates and remapping with MAQ [24], mean coverage of targeted nuclear bases was 37×, 51× and 51× for the three samples. Coverage of ≥8× and a consensus quality score ≥20 was observed for 96%, 94% and 94% of target bases in the nuclear genome. Because of variable coverage or mappability issues with short reads, a small fraction of targeted bases (4 to 6%) were not covered sufficiently to variant call.

**Table 2:** Specificity and depth of coverage for targeted regions

Sample	Sequence output	Percentage of reads mapping to targeted nuclear genes	Percentage of reads mapping to mtDNA genome	Mean fold-coverage of targeted nuclear genes	Mean fold-coverage of mtDNA genome	Percentage of called variants in dbSNP 129
HapMap	356 Mb	17	37	37×	5,001×	90
Patient 1	297 Mb	35	20	51×	2,936×	94
Patient 2	333 Mb	30	27	51×	4,236×	93

Because sample complexity was clearly not limiting for reads mapping to the mtDNA, all reads mapping with MAQ were considered (that is, without removing potential PCR duplicates). Considering only high confidence placements and base qualities (those with both a MAQ mapping score of at least 20 and a MAQ base call quality score of at least 20), mean coverage of the 16,569-bp mitochondrial genome was 5,001×, 2,936×, and 4,236× for the three samples.

### Mutations and new variants of unknown significance

The known mutations and novel non-synonymous variants identified in the study are listed in Table 3. Mutations identified in the two patient samples corresponded to those previously detected by Sanger sequencing. Patient 1 is a male hemizygote for the common mutation R263G in the X-linked alpha subunit of the E1 enzyme (encoded by *PDHA1*) of the Pyruvate dehydrogenase complex (49 reads covered this region and all contained the variant). This nuclear-encoded mitochondrial matrix enzyme complex provides the primary link between glycolysis and the tricarboxylic acid cycle by catalyzing the irreversible conversion of pyruvate into acetyl-CoA. The mutations in patient 2 affected the alpha subunit of the mitochondrial trifunctional protein Hydroxyacyl-CoA dehydrogenase (encoded by *HADHA*), also called long-chain hydroxyacyl-CoA dehydrogenase (*LCHAD*). *LCHAD *deficiency (OMIM 609016) is a mitochondrial autosomal recessive disorder characterized by early-onset cardiomyopathy, hypoglycemia, neuropathy, pigmentary retinopathy, and sudden death due to the defect in the beta-oxidation of fatty acids. Patient 2 is a compound heterozygote for a novel mutation affecting the G nucleotide of the conserved splicing acceptor site [26] at the 5' end of exon 5 (35 reads, 18 with the mutation), and the common mutation E510Q [27] (64 reads, 39 with the mutation).

**Table 3:** New variants and mutations identified in the samples

Alterations	OMIM number	Prediction	Notes
**HapMap sample**			
***PREPL ***[Genbank:NM_006036.3]: c.1769A>C (p.Asn590Ser) het	606407	Possibly damaging*	Same variant present in orthologue Protease II [NP_360014]. *PREPL *was reported as one of the genes deleted in the homozygous 2p21 deletion syndrome
***FXN ***[Genbank:NM_000144.3]: c.626A>G (p.Asp209Gly) het	229300	Possibly damaging*	The *FXN *gene encodes the protein Frataxin, which is involved in mitochondrial iron metabolism. Clinical significance is unclear as this amino acid is not conserved in orthologues. Gly in mouse orthologue
***DBT ***[Genbank:NM_001918.2]: c.725C>A (p.Ser242Stop) het	248600		The *DBT *gene encodes the E2 component of branched-chain alpha-keto acid dehydrogenase complex involved in the catabolism of the branched-chain amino acids. Nonsense mutation at position 224 in single nucleotide polymorphism rs74103423
***MRPL46 ***[Genbank:NM_022163.3]: c.107C>T (p.Ala36Val) het		Benign*	Component of the large subunit of the mitochondrial ribosome. No mutations were reported in patients
***SLC25A45 ***[Genbank:NM_182556.2]: c.299T>C (p.Met100Thr) het		Benign*	Variant in pseudogene [NW_923184.1]. Thr in mouse orthologue
***SLC25A3 ***[Genbank:NM_213611.2]: c.1066A>C (p.Lys356Gln) het	610773	Benign*	Mitochondrial phosphate carrier deficiency can be caused by mutation in the *SLC25A3 *gene, which encodes the mitochondrial phosphate carrier. Variant in pseudogene [NT_009775.16]. Gln in Armadillo orthologue
***PAH ***[Genbank:NM_000277.1]: c.500A>G (p.Asn167Ser) het	261600	Benign*	*PAH *encodes Phenylalanine hydroxylase. This variant was reported as a potential mutation for phenylketonuria [34]
			
**Patient 1 sample: pyruvate dehydrogenase deficiency**			
***PDHA1 ***[Genbank:NM_000284]: c.787C>G (p.Arg263Gly)	312170	**Mutation^†^**	The *PDHA1 *gene encodes the alpha subunit of Pyruvate decarboxylase, the first of three enzymes in the Pyruvate dehydrogenase complex
***MTG1 ***[Genbank:NM_138384.2]: c.151T>G (p.Cys51Gly) het		Probably damaging	*MTG1 *encodes a conserved protein required for assembly of the large ribosomal subunit. Glycine in this position in ribosomal biogenesis GTPase of *Mycoplasma pneumoniae *[NP_110345]. No mutations were reported in patients
***SLC25A5 ***[Genbank:NM_001152.3]: c.811T>C (p.Phe271Leu) het		Possibly damaging	ADP/ATP translocase. Variant in pseudogene [NW_923184.1]
***MRPL9 ***[Genbank:NM_031420.2]: c.637A>G (p.Ile213Val) het		Benign*	Component of the large subunit of the mitochondrial ribosome. No mutations have been reported in patients
***HADHB ***[Genbank:NM_000183.2]:c.3G>T (p.Met1Ile) het	609016	Benign*	The *HADHB *gene encodes the beta subunit of the mitochondrial trifunctional protein involved in mitochondrial beta-oxidation of fatty acids. This variant was not confirmed by Sanger sequencing. Visual inspection of the reads confirmed the Sanger sequencing results
***PCK2 ***[Genbank:NM_004563.2]: c.1470+2T>C het	261650		*PCK2 *encodes Phosphoenolpyruvate carboxykinase 2. Mutations in this gene cause phosphoenolpyruvate carboxykinase deficiency
			
**Patient 2 sample: long chain acyl-CoA dehydrogenase deficiency**			
***HADHA ***[Genbank:NM_000182.4]: c.1528G>C (p.Glu510Gln) het	609016	**Mutation^†^**	The *HADHA *gene encodes the alpha subunit of the mitochondrial trifunctional protein involved in mitochondrial beta-oxidation of fatty acids
***HADHA ***[Genbank:NM_000182.4]: c.315-1G>A het	609016	**Mutation^†^**	
***SLC25A15 ***[Genbank:NM_014252.3]: c.269A>T (p.Gln90Leu) het	238970	Benign	Hyperornithinemia-hyperammonemia-homocitrullinuria syndrome is caused by mutations in the *SLC25A15 *gene, which encodes the mitochondrial ornithine transporter. Variant in pseudogene [NW_923184.1]
***MRPS5 ***[Genbank:NM_031902.3]: c.851G>A (p.Arg284Gln) het		Possibly damaging	Component of the small subunit of the mitochondrial ribosome. No mutations were reported in patients. This position is conserved but not invariant in *MRPS5 *orthologues
***PREPL ***[Genbank:NM_006036.3]: c.1769A>C (p.Asn590Ser) het	606407	Possibly damaging*	Notes as above
***DBT ***[Genbank:NM_001918.2]: c.725C>A (p.Ser242Stop) het	248600		This variant is present in the HapMap sample above
***PAH ***[Genbank:NM_000277.1]: c.500A>G (p.Asn167Ser) het	261600	Benign*	Notes as above
***MRPL9 ***[Genbank:NM_031420.2]: c.637A>G (p.Ile213Val) het		Benign*	Notes as above

GenBank accession numbers are given in square brackets. Polyphen predictions are not available for stop variants or splice site variants. *Variant also seen in normal samples in [23]. ^†^Mutations previously identified in positive controls. Het, heterozygote.

In the three samples, approximately 90% (301 over 336 total variants identified), 94% (297 over 315), and 93% (291 over 314) of the identified variants were previously documented in dbSNP (version 129). A limited number of novel variants were non-synonymous and all in the heterozygote state. Many of the same variants were also identified in unrelated samples from a human exome study that included 12 subjects (Table 3) [23]. The new variants were analyzed with PolyPhen [25], searched in Cardiff's Human Gene Mutation Database [28], aligned in search of homologous regions by BLAST [29] and compared to orthologues with the Conserved Domain Database [30] (Table 3). Only one variant was predicted as probably damaging. This was a cysteine to glycine substitution in the protein encoded by *MTG1*, a conserved protein required for assembly of the large ribosomal subunit [31]. However, an alignment to orthologues showed that non-polar neutral residues can be substituted at this position. In particular, a glycine occupies this position, within a conserved region, in a ribosomal biogenesis GTPase from *Mycoplasma pneumoniae *[GenBank:NP_110345.1], indicating that the observed substitution may be tolerated. Nonetheless, it would be interesting to test the ability of the variant protein to rescue the respiratory deficient yeast *mtg1 *mutant [31], as this may be one of the as yet unidentified causative genes that are present in the population. A novel non-conservative substitution from asparagine to glycine was observed in the penultimate amino acid of Frataxin, a protein involved in the regulation of mitochondrial iron content mutated in one form of Frederich Ataxia (OMIM 229300). This was predicted as a possibly damaging variant. However, this position is not conserved between orthologues and is glycine in mouse, indicating that this terminal amino acid may not be functionally important [32]. Two samples shared a conservative substitution from arginine to serine in Prolyl endopeptidase-like (*PREPL*), a novel oligopeptidase involved in hypotonia-cystinuria syndrome [33]. This was predicted to be possibly damaging; however, in a search of orthologue proteins, a protease from *Rickettsia conorii *[GenBank:NP_360014] was shown to contain serine at the same position within a shared conserved motif. An arginine to glutamine substitution in the protein encoded by *MRPS5*, a member of the small mitochondrial ribosome subunit, was predicted to be possibly damaging. This position is conserved but not invariant in *MRPS5 *orthologues. Phosphoenolpyruvate carboxykinase 2 (*PCK2*) presented a substitution at the donor splice site of intron 9 from the consensus GT to the non-canonical GC. Since GC is observed in some intron donor sites, it is hard to predict if this variant may affect splicing. A missense variant of the first codon of the beta subunit of the mitochondrial trifunctional protein Hydroxyacyl-CoA dehydrogenase (*HADHB*) could not be confirmed with traditional Sanger sequencing. This is an homozygote duplication of CTA in the first exon of the *HADHB *gene that we saw previously in normal samples ([GenBank:NM_000183.2] c.8_10dupCTA) and was also detected in [23]. We then visually inspected the reads and were able to recognize that the variant was actually sequenced properly, while the artifactual variant had been called by MAQ. We believe these artifacts can be reduced with an improved recognition of indel variants using 76-bp reads and utilizing other analysis tools, such as 'cross-match', as exemplified in [23].

Interestingly, both the HapMap individual and patient 2 are carriers for two identical mutations in recessive genes. The first is a novel stop mutation in the gene *DBT*, encoding the Dihydrolipoyl transacylase subunit (E2) of Branched-chain alpha-keto acid dehydrogenase complex, one of the genes causing maple syrup urine disease (OMIM 248600); the second is a known mutation in Phenylalanine hydroxylase (*PAH*) [34], the gene mutated in phenylketonuria (OMIM 261600). This specific variant was observed in one case with benign persistent hyperphenylananinemia, although not conclusively identified as pathogenic [34]. This variant was also identified in normal samples in a human exome study that included 12 subjects, indicating that it is likely a polymorphism [23]. While phenylketonuria is not a mitochondrial disorder, *PAH *was included in the list of candidate genes relying on an approach that uses shared evolutionary history to identify functionally related components of complex I [35]. Additional variants were predicted to be benign. Alignment to the human genome by BLAST identified some of the same variants in pseudogenes; therefore, it is possible that these highly homologous regions were captured as well.

In the HapMap individual and patients 1 and 2 known polymorphisms were also recognized at 41, 28, and 24 positions, respectively, in mtDNA in homoplasmic state (defined here as >95% of high-quality bases corresponding to a non-reference allele at a given position in a given individual). Our criteria for identifying sites of potential heteroplasmy in the mitochondrial genome included >200× coverage of the position with high-quality bases, and the observation of more than one allele at >5% frequency (that is, at least ten high-quality observations of the alternative allele). Six candidate heteroplasmic polymorphisms were identified in the three samples. However, after manual curation, variants in low complexity regions or regions with genome homology could not be confidently called. For example, one variant, m.4716C>A (AC_000021, Revised Cambridge Reference Sequence for mtDNA), was observed in all samples and accounted for 6%, 15% and 21% of the reads in the HapMap and patient 1 and 2 samples, respectively. This is a non-synonymous variant in the gene *ND2*, which encodes the NADH dehydrogenase 2 subunit of complex I. This C>A transversion would cause the missense mutation Gln83Lys in *ND2*, predicted to be probably damaging by PolyPhen. While it has been shown that heteroplasmic pathogenic mtDNA mutations are common in the general population [1], this is likely not a significant variation given the patient's clinical diagnosis. A likely explanation is that a highly homologous pseudogene on chromosome 1 ([GenBank:LOC100131754]; similar to NADH dehydrogenase subunit 2) is also being captured to some extent, and incorrect mapping of some percentage of reads results in the observation of apparent heteroplasmy in all samples. By contrast, a heteroplasmic variant in which we had more confidence, m.16175A>G, was observed in the patient 2 sample, with approximately 50% of reads corresponding to each variant. This position is A in a non-coding region of the reference mtDNA sequence while it is G in a deposited mtDNA sequence (AF346989). We confirmed by Sanger sequencing the presence of this heteroplasmic variant.

## Discussion

We have developed an assay to streamline the molecular diagnosis of mitochondrial disorders by simultaneous sequencing of the entire mtDNA genome and the exons of 362 nuclear genes for targeted mitochondrial proteins. The current list of targeted genes includes 104 nuclear genes for which the causative mutations were previously found in various symptomatic patients [6,8,21,36-40], the entire mtDNA genome, and 258 additional nuclear genes potentially involved in mitochondrial disorders but that were never reported in patients due to either no attempts to sequence them or lack of clinically available testing (Table 1). The known/candidate genes include all of the structural components of oxidative phosphorylation complexes, as well as other mitochondrial proteins of the following functional groups: respiratory complex assembly factors, transcription and translation factors, enzymes, and carrier proteins. Some of the genes causing secondary inhibition of the mitochondrial respiratory chain are also included in this panel. One criterion for inclusion in the list of candidate genes was that members of each group had already been implicated in mitochondrial disease. Some candidate genes were recently reported as components of mitochondrial respiratory complexes by proteomics [6,35] or identified as candidate genes by integrative genomics [6,41]. Since we first compiled the list of putative genes, three were identified as causing mitochondrial disease in patients (*C20orf7*, *CoQ9 *and *NDUFAF3 *[41-43]). This encourages us to interrogate candidate genes in suspected patients with unknown molecular defects.

In order to build a cost-effective but comprehensive diagnostic approach, we performed multiplex capture of the regions of interest using patients' DNA followed by sequencing with an Illumina Genome Analyzer. Considering that the majority of pathogenic mutations are in coding regions or at intron-exon boundaries, we restricted capture and sequencing to these subsequences in genes of interest. The total target size is approximately 0.6 Mb for the exons of the 362 nuclear genes and 16.6 Kb for the entire mtDNA genome. This strategy allows circumventing the high costs of PCR and conventional sequencing for a large number of targets, while maintaining high sensitivity and specificity for detection of potentially pathogenic variants. Coverage of ≥8× and a consensus quality score ≥20, which in our experience allows reliable variant calling [23], was observed for 96%, 94% and 94% of target bases in the nuclear genome in the HapMap, patient 1 and 2 samples, respectively. Normal and patient DNA samples with known pathogenic mutations were tested blindly. All known mutations in two different genes in the patients' DNA samples were identified correctly. The common mutation R263G in the X-linked gene *PDHA1*, which encodes a subunit of the Pyruvate dehydrogenase complex, was identified in the patient 1 sample. The observed mutation in *PDHA1 *has been described in patients with Leigh syndrome [44], a condition characterized by extensive genetic heterogeneity, since it can be due to mutations in several genes (OMIM 256000) and is thus a paradigm for the utility of the proposed assay. The mutations in the patient 2 sample affect *HADHA*, also called long-chain hydroxyacyl-CoA dehydrogenase (*LCHAD*). Patient 2 is a compound heterozygote of a novel mutation affecting the G nucleotide of the conserved splicing acceptor site [26] at the 5' end of exon 5 and the common mutation E510Q [27]. Several polymorphisms in mtDNA were identified and the depth of sequencing coverage was extremely high, indicating that it will be feasible to detect pathogenic mtDNA mutations in the presence of low level heteroplasmy undetectable with Sanger sequencing. While validation with a larger panel of positive controls for nuclear and mtDNA mutations is needed, this approach appears highly promising since approximately 95% of the targeted regions of 362 nuclear genes were sequenced and the results for known mutations were 100% concordant. The high sensitivity of this method as well as the power to identify gene-disease relationships has been well demonstrated in a whole exome sequencing study [23].

While the mutations in the analyzed samples were known at the offset, this study exemplifies the necessity to interpret and validate with traditional sequencing potentially pathogenic new variants identified in patients with unknown molecular defects. However, our results indicate that the number of new variants is not as high as we anticipated. Indeed, of the variants identified in the samples, 90 to 94% were present in dbSNP while 6 to 10% represented new variations. Most of the non-synonymous new variants were predicted to be benign when analyzed with PolyPhen. Only few of the variants were predicted as possibly or probably damaging. However, a review of the literature or alignment to orthologues indicates that these may be tolerated changes. Moreover, after filtering these variants with the new variants identified in normal samples by exome sequencing [45], only three variants in the patient 1 sample and two variants in the patient 2 sample would have required careful interpretation.

In summary, we anticipate that with more data on individual genomes/exomes, the panel of polymorphisms present in the population will grow, thus reducing the need to interpret new variants and the extent of traditional sequencing to confirm the variants. While the use of prediction tools and analysis of the literature on the affected proteins can provide a relatively easy way to assess the significance of the new variants, integrated bioinformatic support seems very important for the successful implementation of next-generation sequencing in the clinical arena.

A variable range of coverage was achieved across the targeted areas and, for this reason or because of the challenges of mapping short reads to the human genome, a small portion of targets (4 to 6%) was not sufficiently covered by sequence reads for variant calling. This aspect will definitely require further improvements that may be achieved with modifications to capture as well as other steps. However, given the number of target genes analyzed and the lack of clinical testing, our initial results are highly encouraging. While it is ideal to achieve coverage for all the targeted regions, this may not be realistic as some regions may be refractory to capture, amplification or sequencing. We plan to expand the target pool to include additional known and candidate genes [9] and also to test the performance of other capture systems [16,46].

A final consideration concerns the applicability of the technique in a clinical setting based on ease of workflow and economic aspects. The sample preparation and set-up of the sequencing runs, while requiring expert handling, is fairly straightforward. Only one sequencing lane was utilized per sample and up to eight samples can be analyzed in one run. Several aspects of the procedure are rapidly improving, allowing increases in sequence output, sample multiplexing, and better data analysis, which will certainly enable a cost-effective approach to the diagnosis of several complex genetic diseases.

## Conclusions

Our data demonstrate that the use of next-generation sequencing holds great promise as a tool for screening mitochondrial disorders in patients. The availability of a diagnostic test will provide opportunities to identify patients early in life, eliminate lengthy and often invasive procedures, and provide life-saving therapies, permitting prompt management and accurate genetic counseling. Furthermore, the ability to diagnose patients will stimulate the development of new targeted therapies based on the known genetic defect. We expect that the analysis of samples from patients with uncharacterized molecular defects will allow the discovery of novel mutations in the targeted candidate genes, thus expanding and redefining the spectrum of mitochondrial disorders.

## Abbreviations

CCDS: Consensus CDS; mtDNA: mitochondrial DNA.

## Competing interests

The authors declare that they have no competing interests.

## Authors' contributions

VV conceived of the study, carried out the sample preparation, analyzed data and drafted the manuscript. SBN and EHT designed reagents and protocols and performed experiments. JS analyzed the data and helped to draft the manuscript. SH conceived of the study, analyzed the data and helped to draft the manuscript. All authors read and approved the final manuscript.
